# Brief Screening of Vascular Cognitive Impairment in Patients With Cerebral Autosomal-Dominant Arteriopathy With Subcortical Infarcts and Leukoencephalopathy Without Dementia

**DOI:** 10.1161/STROKEAHA.116.013761

**Published:** 2016-09-26

**Authors:** Rebecca L. Brookes, Matthew J. Hollocks, Rhea Y.Y. Tan, Robin G. Morris, Hugh S. Markus

**Affiliations:** From the Department of Clinical Neurosciences, University of Cambridge, Addenbrooke’s Biomedical Campus, United Kingdom (R.L.B., M.J.H., R.Y.Y.T., H.S.M.); and Department of Psychology, Psychology and Neurosciences, Institute of Psychiatry, King's College London, London, United Kingdom (R.G.M.).

**Keywords:** activities of daily living, CADASIL, cerebral small vessel disease, cognition, stroke

## Abstract

Cerebral autosomal-dominant arteriopathy with subcortical infarcts and leukoencephalopathy (CADASIL) is a monogenic form of cerebral small vessel disease leading to early-onset stroke and dementia, with younger patients frequently showing subclinical deficits in cognition. At present, there are no targeted cognitive screening measures for this population. However, the Brief Memory and Executive Test (BMET) and the Montreal Cognitive Assessment (MoCA) have shown utility in detecting cognitive impairment in sporadic small vessel disease. This study assesses the BMET and the MoCA as clinical tools for detecting mild cognitive deficits in CADASIL.

**Methods—:**

Sixty-six prospectively recruited patients with CADASIL, and 66 matched controls completed the BMET, with a subset of these also completing the MoCA. Receiver operating characteristic curves were calculated to examine the sensitivity and specificity of clinical cutoffs for the detection of vascular cognitive impairment and reduced activities of daily living.

**Results—:**

Patients with CADASIL showed more cognitive impairment overall and were poorer on both executive/processing and memory indices of the BMET relative to controls. The BMET showed good accuracy in predicting vascular cognitive impairment (85% sensitivity and 84% specificity) and impaired instrumental activities of daily living (92% sensitivity and 77% specificity). The MoCA also showed good predictive validity for vascular cognitive impairment (80% sensitivity and 78% specificity) and instrumental activities of daily living (75% sensitivity and 76% specificity). The most important background predictor of vascular cognitive impairment was a history of stroke.

**Conclusions—:**

The results indicate that the BMET and the MoCA are clinically useful and sensitive screening measures for early cognitive impairment in patients with CADASIL.

Cerebral autosomal-dominant arteriopathy with subcortical infarcts and leukoencephalopathy (CADASIL) is a monogenic form of cerebral small vessel disease (SVD) caused by mutations in the *Notch3* gene, and it is associated with recurrent lacunar strokes and cognitive decline leading to dementia.^1^

Even before the onset of stroke and dementia, cognitive deficits are detected in patients with CADASIL,^2^ particularly in areas of attention, processing speed, and executive functions.^2–5^ Many studies have shown a similar pattern to older patients with sporadic SVD,^6^ although some studies have also shown early deficits in memory retrieval.^3^

Cognitive screening provides clinicians with important information about disease progression and cognitive disability as well as providing rapid measures for use in clinical trials. However, there are few targeted screening measures for SVD in general or, in particular, for patients with CADASIL where deficits may be subtle. Measures developed for cortical dementias such as the Mini-Mental State Examination^7^ and Alzheimer's Disease Assessment Scale- Cognitive Subscale test^8^ are relatively insensitive to subtle changes in key areas of function in SVD, namely executive function and information processing speed because of their lack of adequate measurement of these processes.^6,9^ An alternative is the Brief Memory and Executive Test (BMET), an open access brief screening tool specifically designed for the detection of cognitive deficits in SVD,^10,11^ which has been extensively validated in patients with sporadic SVD. The Montreal Cognitive Assessment (MoCA) may also be a sensitive measure, and it has been validated in a general stroke population^12^ and been shown to be associated with subcortical white matter disease.^13^

The primary aim of our study was to assess the use of the BMET and the MoCA as a rapid screening tool for the detection of vascular cognitive impairment (VCI) and cognitive disability in CADASIL. The study also examines important risk factors and background variables in relation to VCI.

## Methods

### Participants

Sixty-six patients (mean age=51.6, SD=9.5, range=34–70; sex: male=38%) with a genetically confirmed diagnosis of CADASIL, based on a typical disease-causing cysteine altering mutation in the *Notch3* gene, were recruited prospectively from 2 national CADASIL referral clinics at St. George’s Hospital, London, United Kingdom and Addenbrooke’s Hospital, Cambridge, United Kingdom. None of the patients had a clinical diagnosis of dementia. All had brain magnetic resonance imaging.

Five hundred and two healthy controls were recruited from family doctor practices or other volunteer groups in South London as part of the previous BMET validation study.^11^ Individuals with a history of stroke, transient ischaemic attack, major central neurological or major psychiatric disease were excluded. An age- and sex-matched control population (n=66) were randomly selected from this larger sample. Random sampling was conducted in *R* (10^5^ iterations) with the probability of being selected weighted for sex. Controls older than 70 (n=167) years were removed. There were 9 resultant samples closely matching the CADASIL group for the age and sex ratio. A random number generator then selected sample No. 6 from these (age=mean=51.6, *D*=9.0, range=36–70; sex=male=36%). Full details of participant demographics are presented in Table 1.

**Table 1.**
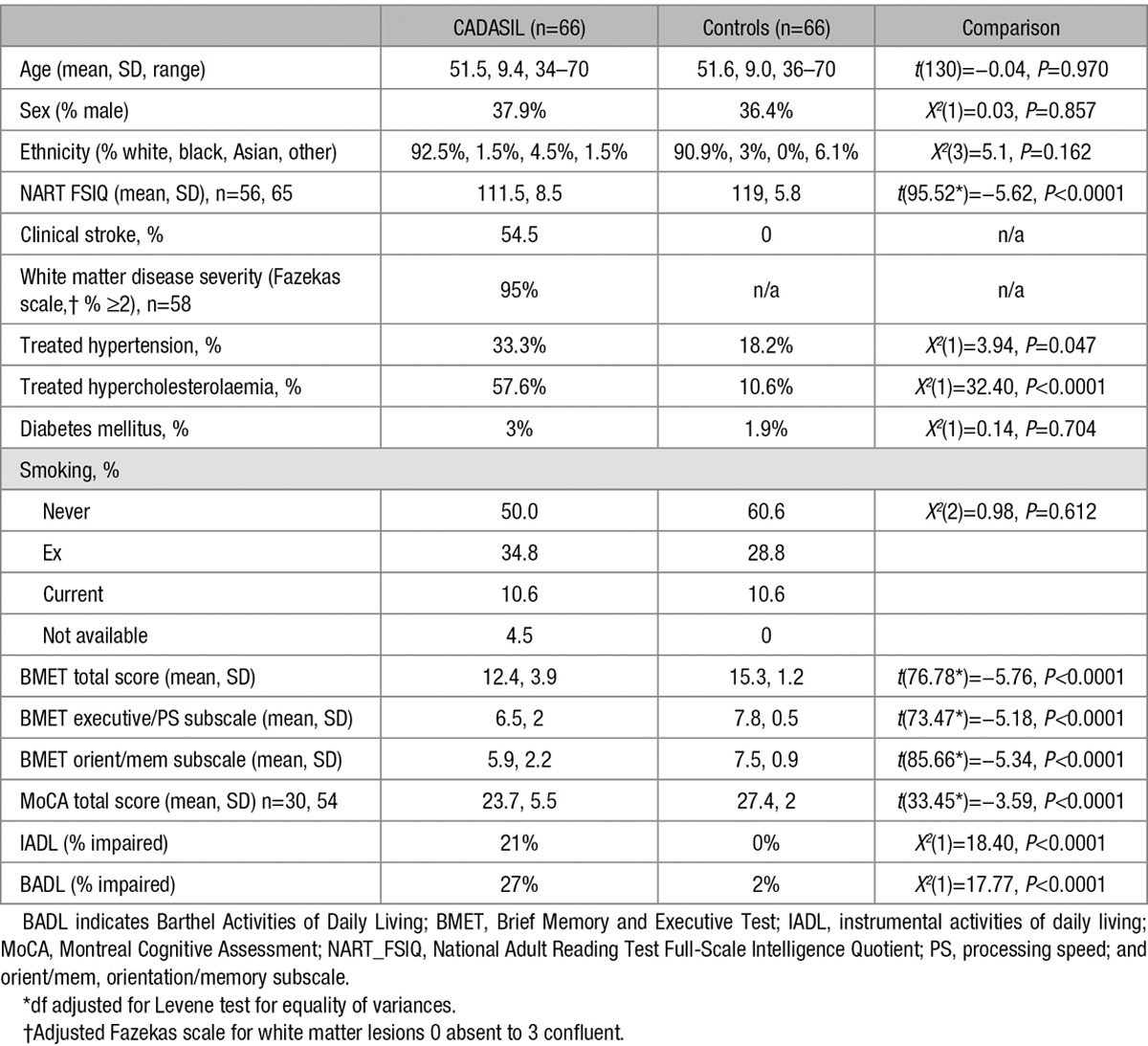
Participant Demographics and Neuropsychology Scores

### Ethics

Participants were recruited as part of studies approved by United Kingdom National Health Service ethics committees. All participants gave informed consent.

### Cognitive Screening Measures

The BMET is a fully normed screening test developed for the detection of VCI.^10,11^ It takes around 10 minutes to administer and contains 4 items sensitive to executive dysfunction/processing speed: (1) letter-number matching, (2) motor-sequencing, (3) letter-sequencing, and (4) number-letter sequencing; and 4 items sensitive to memory impairment: (1) orientation, (2) 5-item repetition, (3) 5-item recall, and (4) 5-item recognition. Raw scores are converted into age-scaled scores of 0, 1, or 2. The total BMET score is out of 16 with a cutoff of ≤13 having previously been shown to indicate impairment.^11^ For participants aged 34 to 39 years, aged 40 test norms were applied. Recent normative data for BMET can be found at http://www.bmet.info. Background interviews and testing were performed in 1 hour research windows to allow maximum participation of patients who were not local to the clinic location in which they were recruited and tested, also reflecting neurological clinic involvement. Where time was available, participants also competed other background measures and the MoCA (n=84).^14^ The MoCA contains 8 sections developed to be relevant to mild cognitive impairment and validated in stroke: (1) visuospatial/executive, (2) naming, (3) memory, (4) attention, (5) language, (6) abstraction, (7) delayed recall, and (8) orientation. The MoCA has a clinical cutoff of <26.

### Additional Measures

The National Adult Reading test (NART-R),^15^ a measure of oral reading vocabulary and premorbid intelligence quotient (IQ; n=121); The Barthel Activities of Daily Living (BADL) scale,^16^ a measure of general disability; and the instrumental activities of daily living (IADL),^17^ a measure of cognitive disability (n=127).

### Statistical Analysis

Demographic variables and cognitive test total scores were compared using *t* tests and χ^2^ tests. To consider the impact of risk factors and background demographics on VCI status in CADASIL, we calculated Pearson χ^2^ for categorical variables and Wald χ^2^ for continuous variables. This was completed for both the MoCA and BMET diagnoses of VCI.

The sensitivity and specificity of the BMET and the MoCA clinical cutoffs for VCI detection was analyzed using receiver operating characteristic (ROC) curves. VCI status was determined using our predefined criteria: modified Petersen mild cognitive impairment threshold of a score of ≤−1.5 SD from the control mean^18^ on ≥4 BMET subtests.^10,11^ To ensure that we did not inflate the effect of the BMET in predicting VCI, we included a second more stringent definition of VCI, which combined both the modified Petersen criteria and the MoCA clinical cutoff for cognitive impairment (<26). To allow for a comparison of the MoCA and BMET, the BMET ROC analysis was rerun in the same population of patients who also had MoCA scores. To further establish predictive validity of the tests, ROC analyses were calculated for an important independent indicator of cognitive disability, impairment of IADL. Reduced IADL was calculated as a score of ≤7 on the IADL, indicating a lack of independence on any of the domains of the IADL. To ensure any prediction of this outcome was not because of general disability, we examined the association with BADL total scores and also clinical impairment on the BADL as defined in the study by Hollocks et al,^19^ using linear and binary logistic regression.

Post-hoc analyses of disease markers and screening test total scores were carried out using linear regression analyses. Post-hoc analyses of the impact of premorbid IQ on screening test scores for detecting VCI were carried out using discriminant function analysis. All analyses were carried out in SPSS (v21).

## Results

### Descriptive Data for Groups

The CADASIL group had significantly lower total scores than controls on the BMET *(t*(76.78)=−5.76, *P*<0.0001). This was also true for both the executive/processing speed subscale (*t*(73.47)=−5.18, *P*<0.0001) and the orientation/memory subscale (*t*(85.66)=−5.34, *P*<0.0001). Using the clinical cutoff of ≤13 on the BMET, the number of patients with CADASIL defined as having cognitive impairment was 32 of 66 compared with 7 of 66 for the controls. For the participants who completed the MoCA, the CADASIL group had significantly lower scores than the controls *t*(33.45)=−3.59, *P*<0.0001). Using the MoCA clinical cutoff of <26, the number of patients with CADASIL defined as having cognitive impairment was 17 of 30 compared with 19 of 54 controls.

### Risk Factors and VCI

For VCI as diagnosed by the BMET, there were no significant effects of hypertension (*X*^*2*^(1)=0.03, *P*=0.862), hyperlipidemia (*X*^*2*^(1)=0.62, *P*=0.432), diabetes mellitus (*X*^*2*^(1)=0.87, *P*=0.350), current smoking status (*X*^*2*^(1)=0.127, *P*=0.722), ethnicity (*X*^*2*^(3)=5.35, *P*=0.148), sex (*X*^*2*^(1)=0.20, *P*=0.655), age (*X*^*2*^(1)=0.005, *P*=0.946), or lesion load (*X*^*2*^(2)=1.57, *P*=0.455). However, there was a significant effect of previous stroke (*X*^*2*^(1)=7.52, *P*=0.006) and also premorbid IQ (*X*^*2*^(1)=9.41, *P*=0.002) on VCI.

For the MoCA diagnosis of VCI, there were no significant effects of any risk factors or background variables: hypertension (*X*^*2*^(1)=0.34, *P*=0.558), hyperlipidemia (*X*^*2*^(1)=0.14, *P*=0.713), diabetes mellitus (*X*^*2*^(1)=0.64, *P*=0.424), current smoking status (*X*^*2*^(1)=0.006, *P*=0.936), ethnicity (*X*^*2*^(2)=3.53, *P*=0.171), sex (*X*^*2*^(1)=0.89, *P*=0.346), age (*X*^*2*^(1)=0.382 *P*=0.537), lesion load (*X*^*2*^(2)=3.07, *P*=0.215), or history of stroke (*X*^*2*^(1)=0.362, *P*=0.547). Premorbid IQ approached significance (*X*^*2*^(1)=2.90, *P*=0.089).

### Markers of Disease and Cognitive Impairment

Post-hoc regression analyses were used to explore further the relationship between disease markers and cognitive impairment. These showed that a history of previous stroke was significantly associated with lower BMET total scores (*β*=−0.378, *P*=0.002), and this held when age was included in the model (*β*=−0.376, *P*=0.004). Fazekas score was not, however, associated with total BMET score (*β*=−0.091, *P*=0.496; with age included: *β*= 0.052, *P*=0.737). The same analyses were carried out for the MoCA. This showed no significant association between a history of previous stroke and MoCA total score (*β*=−0.279, *P*=0.135; with age included in the model: *β*=−0.240, *P*=0.186). Fazekas score was not significantly associated with total MoCA score (*β*=−0.267, *P*=0.170; with age included in the model: *β*=−0.102, *P*=0.607).

### Predicting VCI in CADASIL

On the basis of the modified Petersen criteria, as used in previous analyses,^10,11^ VCI status was given to 24 of 66 patients with CADASIL and 2 of 66 controls. ROC curves were calculated to examine the detection of VCI using the BMET total score. ROC analysis including all participants calculated an area under the curve of 0.94 (95% CI, 0.87–1). A classification of cases based on a BMET cutoff of ≤13 indicated that this criterion had a sensitivity of 85% and specificity of 84% with a total predictive value (TPV) of 84%. This cutoff gave a good balance between sensitivity and specificity. Alternative cut-off points are presented in Table 2. When all controls were treated as unimpaired in the same analysis, a BMET cutoff of ≤13 had a sensitivity of 88% and specificity of 85% with a TPV of 84%.

**Table 2.**
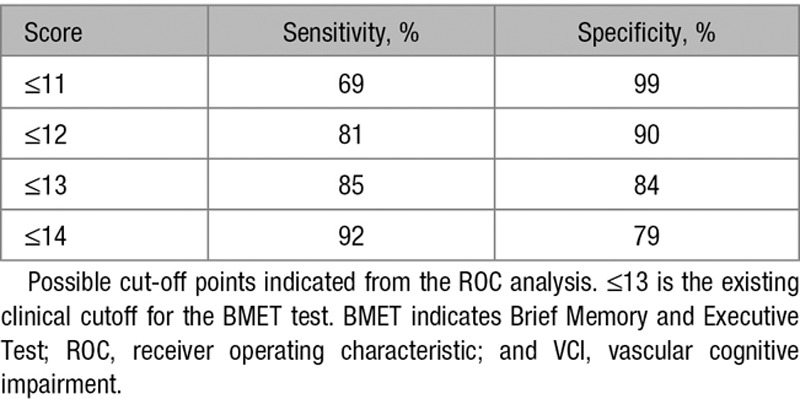
Alternative Clinical Cutoffs for the Prediction of VCI Using the BMET

Eighty-four participants (30 CADASIL and 54 controls) had completed the MoCA. These data were used for a secondary analysis to examine the BMET and the MoCA’s prediction of VCI in parallel. For this subgroup of participants, ROC analysis of the BMET calculated an area under the curve of 0.95 (95% CI, 0.91–1). A classification of VCI cases based on the BMET cutoff of ≤13 gave 81% sensitivity and 88% specificity, with a TPV of 87%. A ROC analysis of the MoCA calculated an area under the curve of 0.87 (95% CI, 0.77–0.97). A classification of VCI cases based on the MoCA clinical criterion of a score of <26 gave 81% sensitivity and 79% specificity, with a TPV of 81%. When all controls were treated as unimpaired using the same analysis, the BMET had a sensitivity of 80% and specificity of 87% with a TPV of 86%, and the MoCA had a sensitivity of 80% and a specificity of 78% with a TPV of 79%.

To avoid bias, adjusted criteria for VCI were created where the participant was defined as impaired if meeting both the original BMET criteria for VCI and the MoCA clinical criterion of <26. The ROC analysis for BMET calculated an area under the curve of 0.99 (95% CI, 0.99–1). A classification of VCI cases based on the BMET cutoff of ≤13 gave 100% sensitivity and 98% specificity, with a TPV of 99%. An ROC analysis of the MoCA calculated an area under the curve of 0.81 (95% CI, 0.67–93). A classification of VCI cases based on the MoCA clinical criterion of a score <26 gave 80% sensitivity and 83% specificity, with a TPV of 82%. When all controls were treated as unimpaired using the same analysis, the BMET had a sensitivity of 100% and specificity of 91% with a TPV of 93%, and the MoCA had a sensitivity of 87% and a specificity of 80% with a TPV of 81%. A summary of the predictive values are given in Table 3.

**Table 3.**
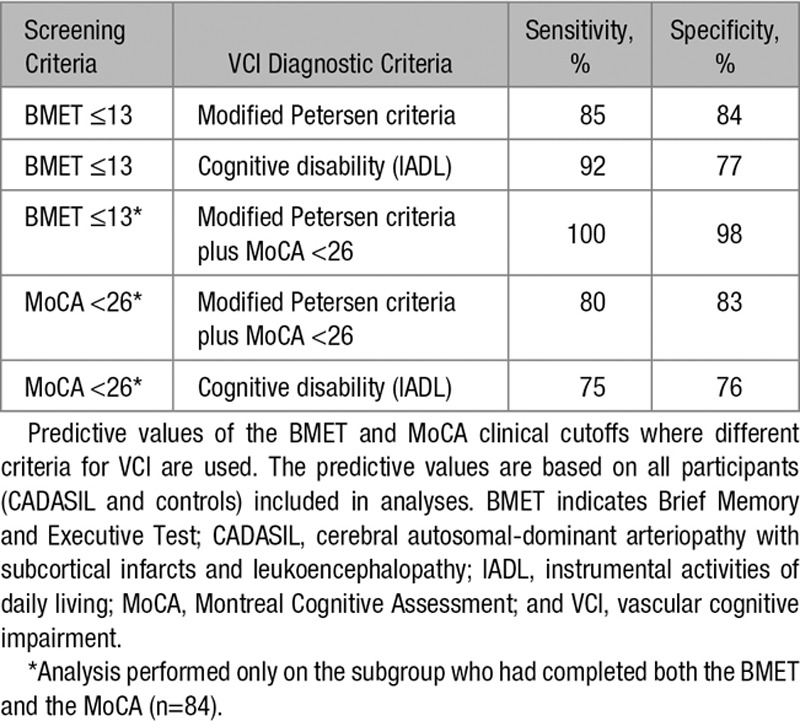
Summary of Predictive Values in for All Participants

### Premorbid IQ and VCI Prediction

Following the finding that premorbid IQ showed a relationship with cognitive test outcomes, we examined whether the addition of premorbid IQ to the screening test cutoffs improved predictive accuracy for VCI. Using discriminant function analyses and adding in premorbid IQ to predict VCI (Petersen criteria), we found that for the whole group sensitivity increased to 91% compared with 85% using the BMET alone and specificity remained the same at 84%. When we took only those participants completing both screening tests, we found that for the BMET plus IQ, sensitivity increased to 92% from 81% and specificity marginally increased to 89% from 88% compared with the BMET alone. Similarly, for the MoCA plus IQ, sensitivity increased to 92% from 81% and specificity increased to 84% from 79% compared with the MoCA alone.

### Predicting Cognitive Disability: Impaired Activities of Daily Living

Using IADL as a marker for cognitive disability, we re-examined the predictive validity of the BMET (n=127; CADASIL=61, controls=66) and the MoCA (n=79; CADASIL=25, controls=54). We found that 13 of 61 patients with CADASIL and 0 of 66 controls in the original sample had cognitive disability. Using ROC analysis to determine the BMETs classification of patients with and without cognitive disability gave an area under the curve of 0.89 (95% CI, 0.78–1.0). The clinical cutoff of ≤13 predicted cognitive disability with a sensitivity of 92%, specificity of 77%, and a TPV of 79%. For the MoCA, the area under the curve was 0.78 (95% CI, 0.53–1.0). The clinical cutoff of <26 predicted cognitive disability with a sensitivity of 75%, specificity of 76%, and TPV of 76%.

For a validity check, we calculated regression models to consider whether the BMET and MoCA’s relationship with the activities of daily living were specific to cognitive disability or a consequence of more general disability in patients with CADASIL. The IADL, an index of cognitive disability, and the BADL, an index of general disability, were included in the models. We found that the BMET significantly predicted IADL (*F*=9.05, df=1, *P*>0.0001) but not BADL total scores (*F*=0.99, df=1, *P*=0.325); and that the MoCA similarly predicted IADL (F=5.63, df=1, *P*=0.026) but not BADL total scores (*F*=0.814, df=1, *P*=0.376), suggesting specificity to cognitive disability. An additional analysis taking clinical impairment as the outcome variable showed the same pattern of results for the BMET (IADL impairment: *X*^*2*^(1)=7.22, *P*=0.007; BADL impairment: *X*^*2*^(1)=0.495, *P*=0.483); the MoCA (IADL impairment: *X*^*2*^(1)=5.46, *P*=0.019; and the BADL impairment: *X*^*2*^(1)=0.053, *P*=0.818).

## Discussion

### Main Findings

On the basis of the modified Petersen criteria,^10,11^ cognitive impairment was found in 24 of 66 patients with CADASIL and 2 of 66 controls. Compared with age-matched controls, the CADASIL group showed a significant reduction in scores on both the BMET and the MoCA and a higher prevalence of VCI.

The primary aim of the study was to look at the use of the BMET and the MoCA, 2 rapid screening measures, in predicting cognitive impairment in CADASIL. The BMET clinical cutoff was derived from normative data used as age-scaled markers by which to compare sporadic SVD cases.^11^ The MoCA clinical cutoff was taken from the test scoring criteria outlined in the manual.^14^ ROC analyses revealed a high predictive value for the BMET clinical cutoff with a TPV comparable to that shown previously in sporadic SVD cases^11^ (CADASIL cases: TPV=84% sporadic cases: TPV=78%). This was maintained across different diagnostic criteria for VCI. The MoCA also performed well. There was, however, a slightly poorer performance than the BMET when both tests were analyzed in parallel, possibly because of the less-sensitive measures of executive function and the lack of processing speed component in the MoCA. In a previous study^11^ of patients with sporadic SVD, the BMET performed well and with similar sensitivity and specificity levels as the current study. However, the MoCA performed less well showing a particularly poor specificity. It may be that the current MoCA cutoff for cognitive impairment over predicts deficits in healthy older adults but be suitable for slightly younger age group, where more subtle deficits are indicative of reduced function. In conclusion, the BMET may have advantages over the MoCA for sporadic, older patients, but not necessarily for younger patients with CADASIL. It should be noted that BMET focuses specifically on executive functioning, processing speed and memory, the reason being that sporadic SVD is particularly associated with executive dysfunction and lower processing speed. The MoCA may detect more widespread cognitive deficit, if it exists, including, for example, visuoconstructional impairment.

In an analysis of the tests' predictive accuracy of cognitive disability, the BMET and MoCA were able to predict, with good accuracy, the existence of cognitive disability as measured by the IADL scale. The data indicated that a quarter of patients completing the IADL scale (13/61) had a deficit affecting their activities of daily living. A reduction in IADLs is strongly associated with reduced cognitive functioning, which leads to impairment in everyday activity.^20^ Of importance is that this study showed a clear and specific prediction of cognitive disability but not general disability. This confirms that these associations were not a consequence of overall disability. Functional disability is an important clinical outcome of CADASIL, and predicting this is important for assessing patient needs. Moreover, these findings were in individuals without diagnoses of dementia, highlighting the need for measures sensitive to the functional impairment seen in this patient group.

### Secondary Analyses

Examining the subdomains of the BMET, significant deficits were seen in both executive functioning/processing speed and also memory. Although it is well established that many patients with CADASIL display deficits in executive functions and processing speed before the onset of dementia,^2,4^ memory deficits have been less commonly identified in previous studies.^3^ They may, however, be worthy of consideration in neuropsychological assessment of this population. Of note is that the distribution of magnetic resonance imaging white matter hyperintensities in CADASIL, although broadly similar to sporadic disease, has more prominent temporal lobe involvement.^21^

Consideration of background variables and risk factors showed that CADASIL patients with a previous stroke were more likely to have a diagnosis of VCI on the BMET than those without. Furthermore, previous stroke predicted overall BMET score even when age was accounted for in the analysis. This is in line with previous research showing executive dysfunction and reduced processing speed of a similar magnitude to sporadic SVD for CADASIL cases with a history of stroke,^6^ and a recent study showed that incident dementia is associated with recurrent stroke in CADASIL.^22^ Fazekas score, however, did not show a significant association with cognitive outcomes on either scale. It is worth noting that this may be because of its relative insensitive to subtle changes in lesion extent, particularly in this population where 95% were graded as 2 or 3. This issue may be better explored in future studies using more quantitative measures of lesion load. Certainly, white matter disease has previously been shown to be associated with the MoCA when using diffusion tensor imaging to map microstructural damage.^13^ Furthermore, the strategic location of white matter damage has also been shown to be of importance when assessing the relationship between lesion and cognition in SVD.^23^ This information is not captured by Fazekas and therefore may add to its insensitivity. Future analyses may need to consider lesion locations in addition to extent.

Our secondary analyses also indicated a significant association between premorbid IQ and cognitive impairment on the BMET, with a borderline association with the MoCA. Premorbid IQ is thought to be associated with predisease neural organization, described as cognitive reserve.^24^ Although reserve in SVD is largely understudied, there is evidence that it might mitigate the relationship between white matter disease and cognitive impairment in normal aging^25^ and also in patients with CADASIL.^26^ Because of this potential influence on cognitive outcomes, we performed a further analysis where premorbid IQ was added into the predictive model for the detection of VCI. Our results showed that even with already high levels of prediction by the BMET and MoCA, the predictive value was further enhanced by the inclusion of premorbid IQ. This emphasises the importance of considering core background variables when performing cognitive screening in patients with SVD and also suggests a potentially fruitful avenue for future research projects looking at cognitive reserve in SVD.

It is acknowledged that these findings need further consideration in larger population. Furthermore, it is noted that the BMET is normed only from age 40 years, and that the use of these norms for those aged <40 (34–39) years may have reduced slightly the levels of impairment in the scores from this group. Furthermore, the lack of normative data for the MoCA may have also reduced its potential sensitivity. A future important direction for cognitive screening measures is their adaptability to younger population.

In conclusion, the results of this study demonstrate that a high frequency of cognitive impairment in nondemented patients with CADASIL and show that the BMET and the MoCA provide a sensitive and specific tools for detecting cognitive deficits in this population.

## Sources of Funding

The BMET study was supported by the Stroke Association (TSA2008/10). R. Brookes is supported by a project grant from the British Heart Foundation (PG/13/30/30005). M. Hollocks is supported by a Stroke Association/British Heart Foundation Grant (TSA BHF 2010/01). R. Tan is supported by the Agency for Science, Technology and Research, Singapore. H. Markus is supported by a National Institute for Health Research Senior Investigator award, and his work is supported by the Cambridge University Hospital Comprehensive National Institute for Health Research Biomedical Research Unit.

## Disclosures

None.
